# IFI16 Mediates Deacetylation of KSHV Chromatin via Interaction with NuRD and Sin3A Co-Repressor Complexes

**DOI:** 10.3390/v17070921

**Published:** 2025-06-28

**Authors:** Anandita Ghosh, Bala Chandran, Arunava Roy

**Affiliations:** 1 Department of Molecular Medicine, Morsani College of Medicine, University of South Florida, Tampa, FL 33620, USA; anandita1@usf.edu (A.G.); chandran@usf.edu (B.C.); 2 Department of Interdisciplinary Oncology, LSU-LCMC Cancer Center, Louisiana State University Health, New Orleans, LA 70112, USA

**Keywords:** IFI16, HDAC1, HDAC2, NuRD, Sin3A, KSHV, herpesvirus, chromatin deacetylation, histone, epigenetics, antiviral restriction factor

## Abstract

IFI16 is a well-characterized nuclear innate immune DNA sensor that detects foreign dsDNA, including herpesviral genomes, to activate the inflammasome and interferon pathways. Beyond immune signaling, IFI16 also functions as an antiviral restriction factor, promoting the silencing of invading viral genes through transcriptional and epigenetic mechanisms. We recently demonstrated another role of IFI16, in which it interacts with and recruits the class I histone deacetylases, HDAC1 and 2, to the KSHV latency protein LANA, modulating its acetylation and function. In this study, we asked whether these IFI16-HDAC1/2 interactions contribute to broader epigenetic regulation of the KSHV chromatin. Our findings reveal that IFI16 associates with and facilitates the recruitment of the NuRD and Sin3A co-repressor complexes—both multiprotein, HDAC1/2-containing chromatin regulators—on KSHV episomes. Depletion of IFI16 led to reductions in NuRD and Sin3A occupancy on viral chromatin, accompanied by increased histone acetylation at lytic gene promoters. These results suggest that IFI16 plays a critical role in recruiting or stabilizing these HDAC-containing co-repressor complexes on the KSHV genome, thereby enforcing transcriptional silencing of lytic genes and maintaining latency in KSHV. Our study expands the known functions of IFI16 and identifies a novel epigenetic mechanism by which it modulates herpesviral chromatin states.

## 1. Introduction

Kaposi’s sarcoma-associated herpesvirus (KSHV), also known as human herpesvirus 8 (HHV-8), is an oncogenic gammaherpesvirus responsible for several aggressive malignancies, most notably Kaposi’s sarcoma (KS), primary effusion lymphoma (PEL), and multicentric Castleman’s disease (MCD) [1,2,3,4,5]. KS is a vascular tumor that predominantly affects immunocompromised individuals, such as those with AIDS, and is thus considered an AIDS-defining illness [6,7,8]. The highest seroprevalence of KSHV is observed in sub-Saharan Africa and certain parts of the Mediterranean [9,10]. In the United States, though KSHV prevalence is estimated at less than 10% in the general population, it can rise to 70% in certain high-risk groups, particularly individuals with HIV/AIDS [11,12]. Currently, there are no specific antiviral treatments available for the management or prevention of diseases associated with KSHV. Available treatment strategies are primarily focused on alleviating symptoms and addressing associated immunosuppression. The absence of targeted therapeutic options underscores the urgent need for in-depth research into the molecular mechanisms underlying KSHV pathogenesis and the regulation of its latency.

Latency plays a central role in KSHV-associated cancers by enabling the virus to persist within host cells while evading immune detection and promoting oncogenesis. Unlike lytic replication, which is both immunogenic and cytopathic, latency enables long-term viral persistence and provides a sustained oncogenic stimulus, making it a critical driver of KSHV-induced tumors [13,14]. During latency, KSHV expresses a limited set of genes, including LANA, vFLIP, and vCyclin, which manipulate host cell cycle, induce angiogenesis, inhibit apoptosis, and drive malignant proliferation [15,16,17,18,19,20,21]. These latency proteins also interfere with key tumor suppressor pathways and promote chronic inflammation, creating a microenvironment conducive to malignant transformation [21,22]. Therefore, a comprehensive understanding of the latency mechanism of KSHV is essential for developing effective therapeutic options.

KSHV establishes and maintains its latency in host cells by tightly regulating its episomal genome through host-derived epigenetic mechanisms [23,24,25,26,27,28]. Upon nuclear entry, the KSHV genome rapidly acquires histones and becomes chromatinized, allowing it to be subject to host chromatin regulatory pathways [29]. During latency, the KSHV episome is marked by repressive histone modifications, including H3K27me3 and H3K9me3, which are deposited by host polycomb repressive complexes and heterochromatin-associated factors, respectively [23,30,31,32]. These modifications, along with other epigenetic mechanisms such as DNA methylation and nucleosome positioning, help silence the lytic gene cascade while maintaining transcription of the latency-associated genes, ensuring a stable latent reservoir of the KSHV genome that retains the capacity for rapid reactivation in response to environmental cues or host stress signals [28,33].

We have previously reported that interferon gamma-inducible protein 16 (IFI16), a ubiquitously expressed and multifunctional host protein, plays a pivotal role in modulating gene expression in herpesviruses, including KSHV and EBV (Epstein–Barr virus) [31,34,35,36]. Beyond its well-characterized function as a DNA sensor in innate immunity—where it detects foreign DNA, such as those from infecting viruses, and subsequently induces inflammasome activation and interferon responses—IFI16 has also been identified as an antiviral restriction factor against several DNA viruses, including KSHV, herpes simplex virus type 1 (HSV-1), EBV, human cytomegalovirus (HCMV), and human papillomavirus (HPV) [23,37,38,39,40]. Two distinct mechanisms have emerged elucidating how IFI16 exerts its restriction factor effects against DNA viruses: (i) as a transcriptional regulator, wherein it modulates transcription factors such as Sp1, Oct1, and TATA-binding protein (TBP) [23,37,41,42]; and (ii) as an epigenetic modulator of viral chromatin [23,31]. Our previous investigations demonstrated that IFI16 interacts with and recruits the histone H3 lysine 9 trimethylation (H3K9me3) methyltransferases SUV39H1 and GLP to KSHV lytic promoters, culminating in their heterochromatization and subsequent silencing [31]. Thus, by linking innate immune sensing to epigenetic silencing pathways, IFI16 serves as a critical regulator at the intersection of host defense and chromatin dynamics.

Recently, we demonstrated that IFI16 also interacts with the class I histone deacetylases (HDACs), HDAC1 and HDAC2, in both KSHV-infected and uninfected cells [43]. We showed that IFI16 recruits these deacetylases to the major KSHV latency protein, LANA, thereby facilitating its deacetylation from its default acetylated state [43]. This deacetylated form is adept at binding to KSHV chromatin, thereby inducing the silencing of key KSHV promoters essential for establishing and maintaining latency. However, the role of this interaction between IFI16 and HDAC1/2 in modulating the epigenetic landscape of the KSHV chromatin still remains an open question. Specifically, it remains unexplored whether this interaction significantly influences the acetylation and deacetylation processes of histones associated with the viral genome. Here, we have elucidated the potential implications of this interaction for the regulation of KSHV chromatin dynamics.

Histone acetylation is a crucial epigenetic modification that profoundly influences chromatin architecture and gene expression. It involves the covalent attachment of acetyl groups to the lysine residues on the N-terminal tails of histone proteins, a reaction catalyzed by histone acetyltransferases (HATs) [44]. The addition of acetyl groups neutralizes the positive charges on histones, which decreases their interaction with the negatively charged DNA [45,46]. Consequently, this results in a more open chromatin structure, which promotes transcriptional activation. Conversely, histone deacetylases (HDACs) are responsible for removing these acetyl groups, leading to a compact chromatin configuration that suppresses transcription. Thus, an intricate balance between histone acetylation and deacetylation is vital for transcriptional regulation.

Here, based on our previous finding that IFI16 interacts with HDACs 1 and 2 [43], we investigated whether IFI16 recruits these class 1 HDACs to the KSHV chromatin, leading to the deacetylation of KSHV chromatin-associated histones. We found that knockdown (KD) of IFI16 resulted in a marked decrease in the abundance of both HDAC1 and HDAC2, accompanied by a significant increase in the levels of acetylated histones, particularly acetyl-H2B, on KSHV promoters. Subsequently, to further elucidate the molecular mechanism of this IFI16-mediated HDAC deposition, we conducted LC-MS/MS protein identification studies to determine whether IFI16 associates with any additional HDAC-recruiting complexes. Our results indicate that IFI16 interacts with the NuRD and Sin3A complexes, both of which are well-established multi-protein repressive complexes that recruit HDAC1 and HDAC2 to the chromatin. Thus, our findings reveal the critical role of IFI16 in recruiting these HDAC-containing co-repressor complexes to the KSHV genome, thereby contributing to the understanding of KSHV’s epigenetic regulation.

## 2. Materials and Methods

### 2.1. Cells

The KSHV-latently infected PEL cell line, BCBL-1, was obtained from the AIDS Malignancy Consortium (AMC). It was cultured in 10% (vol/vol) fetal bovine serum (FBS, Atlanta Biologicals, Oakwood, GA, USA) and penicillin-streptomycin (Gibco Life Technologies, Waltham, MA, USA) supplemented RPMI-1640 medium with GlutaMax (Gibco Life Technologies). TREX-BCBL-1-RTA cells, a kind gift from Dr. Jae Jung, Cleveland Clinic, were cultured in the same medium supplemented with hygromycin B (200 μg/mL). All cell cultures were regularly tested to ensure they were free from mycoplasma contamination using a mycoplasma detection kit (Lonza, Basel, Switzerland).

### 2.2. Lentivirus-Mediated Knockdown of IFI16 in BCBL-1 Cells

To knock down IFI16, we used human TRC short hairpin RNA (shRNA) constructs for IFI16 (TRCN0000019080, TRCN0000019082, TRCN0000019083) from Dharmacon (Horizon Discovery, Waterbeach, UK) and co-transfected HEK293T cells along with lentivirus packaging vectors with CalPhos mammalian transfection kit (TaKaRa, Clontech, Kasatsu, Japan) following the manufacturer’s protocol. To avoid off-target effects, we used a pool of three shRNA clones. We used pLKO.1 empty vector from Dharmacon (Horizon Discovery) as control. 16 h post-transfection, the culture medium was changed, and after 48 h, the supernatants containing the packaged lentivirus particles were collected after filtering through a 0.45 μm filter to discard any cells. The supernatant of all three clones targeting IFI16 was pulled together to transduce the BCBL-1 cells in the presence of 5 μg/mL polybrene.

### 2.3. Mass Spectrometry

Pre-cleared 500 ug of BCBL-1 whole cell lysate (WCL) was immunoprecipitated overnight at 4 °C with the indicated antibodies. Co-IP was performed as mentioned previously [43]. Following Co-IP, the washed beads were subjected to on-bead trypsin digestion following standard protocols. The trypsin-digested peptides were extracted in 50% acetonitrile/0.1% trifluoroacetic acid (TFA) and were characterized using a Thermo Q-exactive-HF-X mass spectrometer coupled to a Thermo Easy nLC 1200 (Thermo Scientific, Waltham, MA, USA). After extraction, samples were separated at 300 nL/min on an Acclaim PEPMAP100 trap (75 μM, 2 cm, c183 μm, 100 A) and an easy spray100 column (75 μm, 25 cm, c18, 100 A) with a 120 min gradient and an initial starting condition of 2% B buffer (0.1% formic acid in 90% acetone) in additional with 98% A buffer (0.1% formic acid in water). After 90 min, the concentration of B buffer was increased to 28% and then up to 40% for an additional 10 min. After which, 90% buffer B was run for 15 min. The mass spectrometer was then equipped with a Thermo nanospray easy source with the following parameters: spray voltage, 1.8; capillary temperature, 250 °C; funnel RF level, 40, and for data acquisition the parameters used were as follows: for MS data, resolution was 60,000 with an AGC target of 3^6^ and a max IT time of 50 ms, the range was set to 400–1600 *m*/*z*. The acquired resolution for MS/MS data was 15,000, with an automatic gain control (AGC) of 1^5^ and a maximum ion time (IT) of 50 ms. The top 30 peaks were picked with an isolation window of 1.6 *m*/*z* and a dynamic execution of 25 s. The acquired data were processed using MaxQuant 2.0.3.1, and peptides were quantified using label-free quantification (LFQ). Reviewed human and the HHV8 virus databases were downloaded from Uniprot (release 2024_06) and searched, keeping the following parameters: a tryptic enzyme with a maximum of two missed cleavages, a fragment mass tolerance of 0.02 Da, and a precursor mass tolerance of 10 ppm.

Protein intensities were log2-transformed and compared between IFI16 IP and IgG controls. Enrichment was assessed using log2 fold change (FC) and unpaired two-tailed *t*-tests (*n* = 2 replicates per condition). Proteins with Log2FC ≥ 0.6 were considered enriched. Statistical analysis and visualization were performed using Python (3.12.0) and R-based tools built on OpenAI’s ChatGPT (GPT-4).

### 2.4. Immunofluorescence Assay (IFA)

BCBL-1 cells were plated on 10-well chamber slides and air-dried. They were then fixed and permeabilized with ice-cold acetone. The permeabilized cells were washed once with PBS and blocked with Image-iT FX signal enhancer (Invitrogen, Carlsbad, CA, USA) for 30 min at 37 °C. After blocking, the cells were incubated for 1 h at 37 °C with primary antibodies specific to the proteins of interest. Then, they were washed three times with PBS and incubated again for 1 h at 37 °C with appropriate fluorescent dye-conjugated secondary antibodies. The slides were then mounted with mounting media containing DAPI (Sigma, St. Louis, MO, USA). The processed slides were observed under a Keyence (Osaka, Japan) BZ-X fluorescence microscope at 60X magnification, and the images were analyzed using the Keyence analyzer software (1.0.1). The experiments were conducted three times independently, and a representative field is displayed.

### 2.5. Proximity Ligation Assay (PLA)

PLA was conducted following the manufacturer’s protocol (DUOLink PLA Kit from Sigma). In short, BCBL-1 cells were grown in chamber slides and then fixed and permeabilized with ice-cold acetone. After washing the acetone with PBS, the cells were blocked with Image-iT FX signal enhancer (Invitrogen) for 30 min at 37 °C, followed by incubation with relevant primary antibodies for 1 h at 37 °C. The cells were then washed with wash buffer A and incubated with the appropriate species-specific plus and minus probes for 1 h at 37 °C. DUOLink antibody diluent buffer was used to dilute the primary antibodies and the PLA probes. Following incubation with the probes, the cells were subjected to a ligation buffer at 37 °C for 30 min, allowing the probes to ligate, followed by two washes with wash buffer A. After ligation, the cells were rewashed with wash buffer A and treated with an amplification-polymerase solution for 1.5 h at 37 °C in a humidified chamber. Following incubation, the cells were washed three times with wash buffer B and then mounted using DUOLink in situ mounting medium with DAPI. PLA signals were detected as distinct fluorescent dots under a Keyence fluorescence microscope at 60 × magnification with an oil immersion objective. PLA signals (dots) from at least 15 cells were counted using ImageJ (1.54) and plotted as a swarm plot using BioRender.com.

### 2.6. Chromatin Immunoprecipitation (ChIP)

Nuclei from BCBL-1 cells were extracted, and their chromatins were sheared using the truChIP chromatin Shearing kit (Covaris, Woburn, MA, USA) following the manufacturer’s protocol on a Covaris ME220-focused ultrasonicator. Following shearing, Triton X-100 and NaCl were adjusted in the sheared lysate to a final concentration of 1% and 150 mM, respectively. The sheared chromatin fragment size was ensured to be between 200 and 500 bps using the 2100 Bioanalyzer instrument and the Agilent High-sensitivity DNA kit (Agilent Technologies, Santa Clara, CA, USA), according to the manufacturer’s protocol. Next, 10 mg of sheared chromatin was incubated overnight at 4 °C with 2 mg of the desired antibody or ChIP-grade control IgG. Next, ChIP-grade protein G magnetic beads (Active Motif, Carlsbad, CA, USA) were added to the chromatin-antibody complex and incubated for 2 h at 4 °C. After immunoprecipitation, the chromatin-antibody-bead complex was washed three times with low-salt and once with high-salt wash buffer (Cell Signaling Technologies, Danvers, MA, USA). To elute the chromatin, the beads were incubated in ChIP elution buffer (Cell Signaling Technologies) at 65 °C for 30 min with shaking at 1200 rpm. The eluted chromatin was treated with NaCl and proteinase K for 2 h at 65 °C to eliminate all protein and reverse the cross-linked protein–DNA interactions. The Immunoprecipitated DNAs were purified using the ChIP DNA Clean and Concentrator Kit (Zymo Research, Irvine, CA, USA) and quantified by qPCR with Power SYBR Green PCR Master Mix (Applied Biosystems, Foster City, CA, USA) using the respective primers (5′ → 3′), pK8 → Forward: GCGTAATTACTTCCGAGACTGA, Reverse: TTAACTCCACTTTGCACCAAAC, and pORF63 Forward: GGGTGTTAGCAGCATATCCATAG, Reverse: CCTCGTGTATTCACAGACCTTTAG. ChIP enrichments were calculated as a percent input relative to input chromatin (2% input) and expressed as fold enrichment over control ChIP.

### 2.7. qRT-PCR

Total RNA extraction was performed utilizing the RNeasy mini kit (Qiagen, Venlo, The Netherlands), followed by on-column DNase digestion using an RNase-free DNase set (Qiagen) in accordance with the manufacturer’s instructions. The concentrations of the extracted RNA were determined using a NanoDrop spectrophotometer, and equal quantities of RNA were employed to synthesize cDNA with the High-Capacity cDNA Reverse Transcription Kit (Applied Biosystems), following the manufacturer’s protocol. The resulting cDNA served as a template for real-time quantitative reverse transcription-PCR (qRT-PCR) of the KSHV ORF25 mRNA using primers: Fwd, 5′-CTCGGCGACGTGCTATACAAT; and Rev, 5′-TGCCGACAAGGACTGTACATG. qPCR was conducted with Power SYBR Green PCR Master Mix (Applied Biosystems) on an ABI Prism 7500 detection system (Applied Biosystems). All RNA levels were normalized to the endogenous controls, specifically RNAaseP, and the quantification was calculated utilizing the ΔΔCT method.

### 2.8. Antibodies

The antibodies used for this study are: IFI16, anti-mouse (Santa Cruz Biotechnology, Dallas, TX, USA, SC-8023); IFI16, anti-rabbit (Millipore Sigma, HPA073514); KSHV LANA, anti-rabbit (In-house); HDAC1, anti-rabbit (Proteintech, Rosemont, IL, USA, 10197-1-AP); HDAC1, anti-goat (Origene, Rockville, MD, USA, TA348968); HDAC2, anti-rabbit (Proteintech, 12922-3-AP); CHD4, anti-rabbit (Cell Signaling, 12011); SIN3A, anti-rabbit (Cell Signaling, 8056); CoREST anti-rabbit (Cell Signaling, 14567); Histone H2A anti-rabbit (Cell Signaling, 12349); Acetyl-Histone H2A anti-rabbit (Cell Signaling, 2576); Histone H2B anti-rabbit (Cell Signaling 12364); Acetyl-Histone H2B anti-rabbit (Cell Signaling, 12799); Histone H3 anti-rabbit (Cell Signaling, 4499); Acetyl-Histone H3 anti-rabbit (Cell Signaling, 8173); Histone H4 anti-rabbit (Cell Signaling, 13919); Acetyl-Histone H4 anti-rabbit (Cell Signaling, 13534); anti-rabbit control IgG (Proteintech, 30000-0-AP); Anti-rabbit IgG Alexa Fluor 488 (Invitrogen, A-11008); Anti-mouse IgG Alexa Fluor 488 (Invitrogen, A-11001).

## 3. Results

### 3.1. IFI16 Modulates Histone Acetylation on KSHV Chromatin

**Figure 1:**
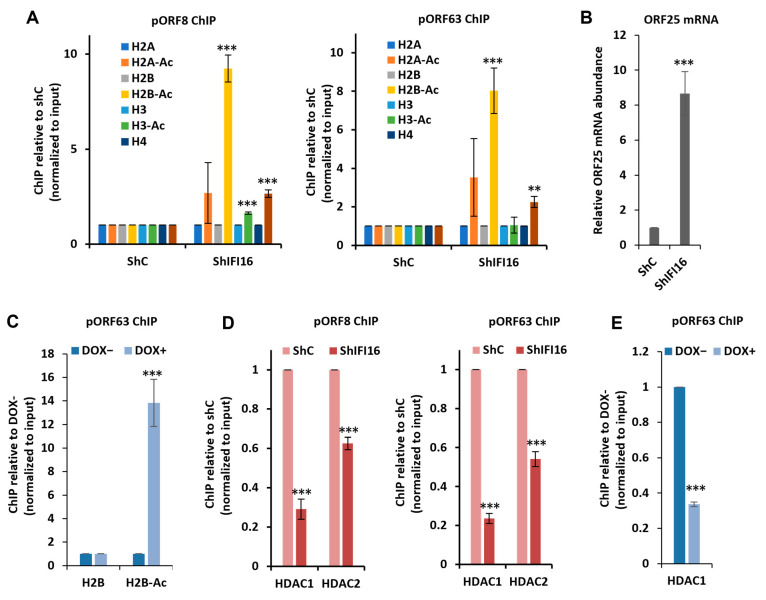
Role of IFI16 in histone acetylation and HDAC recruitment on KSHV chromatin. (**A**) ChIP-qPCR analysis was conducted on core histones (H2A, H2B, H3, and H4) and their acetylated (Ac) forms at the ORF8 and ORF63 promoters (pORF8 and pORF63) of the KSHV genome in BCBL-1 PEL cells transduced with either control shRNA (shC) or shRNA targeting IFI16 (shIFI16) for 72 h. ChIP efficiencies are represented relative to shC, and the acetylated histones have been normalized to their respective non-acetylated forms. (**B**) RT-qPCR of KSHV ORF25, a late gene, 72 h after IFI16 knockdown in BCBL-1. mRNA levels were normalized against RNaseP mRNA and are expressed relative to shC. (**C**) ChIP-qPCR of H2B and acetylated H2B (H2B-Ac) at pORF63 following doxycycline-induced (DOX+) lytic reactivation of TREX-BCBL-1 cells. ChIP efficiencies are represented relative to the uninduced (DOX-), and H2B-Ac has been normalized to its non-acetylated form (H2B). (**D**) ChIP-qPCR of HDAC1 and HDAC2 at pORF8 and pORF63 in BCBL-1 cells after IFI16 knockdown (KD). ChIP efficiencies are represented relative to the shC. (**E**) ChIP-qPCR of HDAC1 occupancy during lytic reactivation (DOX+) of TREX-BCBL-1 cells. ChIP efficiencies are represented relative to the uninduced (DOX−). All error bars represent mean ± standard deviation (SDEV) from three independent biological replicates. Statistical significance was determined by unpaired two-tailed Student’s *t*-test; *p* < 0.01 (**), *p* < 0.001 (***).

To investigate the potential role of IFI16 in modulating the acetylation of histones bound to KSHV chromatin, we first knocked down IFI16 in BCBL-1 (Body Cavity Based Lymphoma-1) cells, a cell line generated from the B cells of a male primary effusion lymphoma (PEL) patient that is latently infected with KSHV and can stably maintain the KSHV episome in culture [47,48]. We lentivirally transduced the cells with shRNA targeting IFI16 (shIFI16) or a non-targeting control (shC) and then performed chromatin immunoprecipitation (ChIP) targeting all four core histones—H2A, H2B, H3, and H4—and their acetylated forms at two KSHV promoters: pORF8 and pORF63. We chose these two promoters based on a previous ChIP-on-chip analysis of the global abundance of H3-Ac (H3K9/K14-Ac) on the KSHV genome in BCBL-1 cells by Günther et al. [49]. These promoters were among the highest H3K9/K14-Ac enriched peaks in this report. Notably, the global abundance of other acetylated histones across the KSHV genome remains unreported to date. Also, to account for changes in the levels of core histones on the KSHV genome in response to IFI16 depletion, we normalized the ChIP abundances of the acetylated histones to those of their unacetylated counterparts. As shown in Figure 1A, we observed that after IFI16 KD, the abundance of all four acetylated histones increased on both the KSHV promoters tested, with H2B-acetylation demonstrating the most pronounced increase. To validate these findings and their alignment with our previously reported observations, indicating that IFI16 KD facilitates lytic reactivation [35], we examined the mRNA expression levels of the late lytic gene, OFR25 (Figure 1B). Our results confirm that IFI16 KD indeed leads to an upregulation of KSHV late lytic gene expression.

It is well accepted that histone acetylation is associated with an open chromatin conducive to transcriptional activity. However, the significance of H2B acetylation in the context of KSHV gene transcription during its latency to lytic switch remains unreported. Thus, to ascertain if the observed increase in H2B acetylation in response to IFI16 KD correlates with the KSHV life cycle, we assayed the abundance of H2B-Ac on the ORF63 promoter during reactivation from latency to lytic cycle. For this, we chose the TRExBCBL-1-Rta cells that carry an epitope-tagged KSHV lytic cycle switch replication and transcription activator (RTA, ORF50) protein cassette under the control of a tetracycline-inducible promoter, so that they can be lytically reactivated using doxycycline (DOX) induced RTA expression [50] instead of chemical inducers like phorbol esters and sodium butyrate, which are known to alter histone acetylation artificially. We observed that 72 h after DOX induction, the abundance of H2B-Ac on the ORF63 promoter increased about 13-fold (Figure 1C). This is not surprising as ORF63 is a lytic gene and is expected to undergo transcriptional activation in response to lytic induction. These findings therefore support the idea that H2B acetylation is associated with active KSHV gene expression. Similar observations have also been reported for H3-Ac and H4-Ac, where increased deposition of these acetylated histones was detected during KSHV reactivation [27,51,52].

Collectively, these findings indicate that IFI16 is essential for the maintenance of KSHV chromatin-bound histones in a deacetylated state during viral latency, which effectively contributes to the silencing of KSHV lytic genes. The depletion of IFI16 disrupts this heterochromatizing environment, resulting in increased histone acetylation at the promoter regions. This observation is consistent with our previous study, which demonstrated that the depletion of IFI16 induces lytic reactivation in latently infected cells, while KSHV lytic reactivation triggers the proteasomal degradation of IFI16 [35].

### 3.2. IFI16 Depletion Reduces HDAC1 and HDAC2 Occupancy on Viral Chromatin

We have previously reported that IFI16 interacts with HDACs 1 and 2 [43]. Therefore, we next investigated whether the depletion of IFI16 would reduce the abundance of these HDACs on the KSHV promoters. ChIP assays similar to the ones described in Figure 1A showed that KD of IFI16 results in the reduced recruitment of both HDACs 1 and 2 on the KSHV ORF8 and ORF63 promoters (Figure 1D). We also probed the abundance of HDAC1 on the ORF63 promoter after lytic induction of TREXBCBL-1 cells. We found that, consistent with the observation in Figure 1C, where lytic induction leads to increased H2B acetylation, the abundance of HDAC1 decreases significantly after reactivation (Figure 1E). Together, our observations suggest that IFI16 plays a role in the recruitment of HDACs 1 and 2 on the KSHV chromatin during latency, and HDAC1 dissociates from the KSHV chromatin during lytic reactivation. This loss of HDAC1 recruitment facilitates chromatin relaxation and viral gene expression.

### 3.3. IFI16 Interacts with Components of the NuRD and the Sin3A Complexes

The established understanding of HDACs indicates that these enzymes lack the inherent capability to directly bind to chromatin-associated DNA or histones independently [53,54]. Instead, each class of HDACs engages with a variety of multiprotein complexes, which are crucial for their recruitment to target chromatin [53,54]. On the other hand, IFI16, which we previously identified as a binding partner for HDACs 1 and 2, can only interact non-specifically with its target foreign DNA, such as the KSHV genome, predominantly through ionic interactions [55,56,57]. Therefore, the likelihood of IFI16 directly recruiting HDACs 1 and 2 to promoters on the KSHV chromatin appears unlikely. However, IFI16 possesses an intrinsic capacity to bind to various cellular proteins via its N-terminal pyrin domain (PYD) [58]. In our preceding study, we demonstrated that IFI16 binds to the KSHV latency protein LANA, facilitating the recruitment of HDACs 1 and 2, thereby promoting its deacetylation [43]. Based on these considerations, we next asked whether IFI16 interacts with any other epigenetic complex that may, in turn, recruit HDACs 1 and 2.

**Figure 2:**
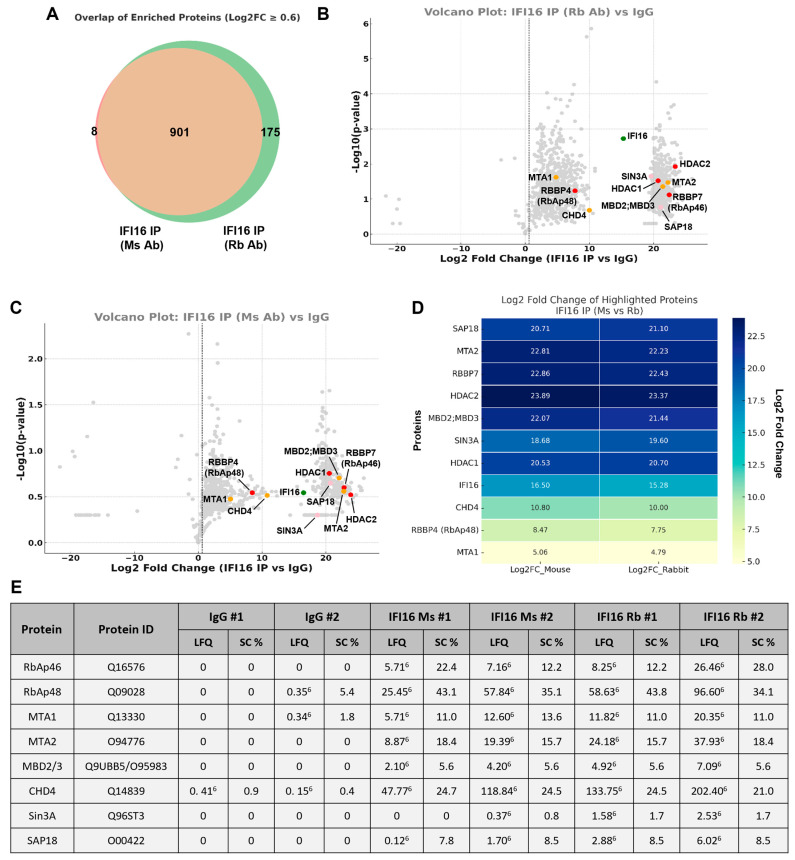
Immunoprecipitation (IP) of IFI16 followed by label-free quantitative mass spectrometry (IP-LFQ-MS) analysis. Whole-cell lysates of latently infected BCBL-1 cells were immunoprecipitated with two separate anti-IFI16 antibodies: anti-IFI16 mouse monoclonal (Ms), anti-IFI16 rabbit polyclonal (Rb), or IgG (Rb), followed by purification of the co-precipitated proteins using Protein A/G Dynabeads (two biological replicates, each). Subsequently, on-bead trypsin digestion and LC-MS/MS were performed to identify the co-precipitated proteins. (**A**) Venn diagram of proteins enriched (Log2 fold change ≥ 0.6) in the two IPs using mouse and rabbit antibodies against IFI16, compared to IgG controls. Proteins common to both the IPs are represented in orange, while those exclusive to the Ms antibody are represented in pink, and those exclusive to the Rb antibody are represented in green. (**B**,**C**) Volcano plot of proteins enriched in IFI16 IP using the Rb antibody (**B**), and the Ms antibody (**C**) compared to IgG controls. Proteins identified by LC-MS/MS were analyzed for differential abundance between IP conditions, and log2 fold change and −log10 *p*-values were calculated for each protein. Highlighted proteins (colored dots) indicate the proteins of interest: Red: core subunit proteins common to both the NuRD complex and the Sin3A complex (HDAC1, HDAC2, RbAp46, and RbAp48). Orange: proteins of the NuRD complex, other than the core subunit (CHD4, MTA1, MTA2, and MBD2/3). Pink: proteins of the Sin3A complex, other than the core subunit (Sin3A and SAP18). Green: IFI16 itself (bait). The dashed lines indicate the Log2FC ≥ 0.6 threshold. (**D**) Heatmap showing the Log2 fold change values for the highlighted proteins of interest, comparing the two IFI16 IPs, Ms and Rb antibodies versus IgG controls. Fold changes were calculated from LFQ intensities using duplicate replicates per condition. Proteins consistently enriched across both antibody conditions support their role as robust interactors of IFI16. (**E**) The label-free quantitation intensities (LFQ) and sequence coverage (SC%) of the proteins of interest.

To address this, we conducted immunoprecipitation (IP) of IFI16, followed by label-free quantitative mass spectrometry (IP-LFQ-MS) to define the protein interaction network associated with IFI16 in BCBL-1 cells. We use two distinct IFI16 antibodies: a mouse monoclonal antibody (Ms) and a rabbit polyclonal antibody (Rb), allowing for the execution of two separate IP experiments, which were individually subjected to MS analysis. As a control for potential non-specific interactions, IgG immunoprecipitations were also conducted. A Venn diagram of proteins enriched above the Log2FC ≥ 0.6 threshold (Figure 2A) showed substantial overlap between these two datasets, supporting the reproducibility and robustness of the IFI16-associated interactome. As illustrated in Figure 2A, we observed a mutually common subset of proteins that were enriched in both IFI16 immunoprecipitations, depicted in orange, while proteins uniquely enriched in the rabbit and mouse antibody IPs are represented in green and pink, respectively. From this analysis, we identified 901 proteins that were present in both the IPs (orange in Figure 2A), likely signifying robust and specific components of the IFI16-associated proteome. We subsequently analyzed the differential abundance of these proteins relative to the IgG pull-down, as shown in Figure 2B,C for the Rb and Ms antibodies, respectively. Interestingly, among the proteins with the highest enrichment within this common set, we identified a complete assembly of individual members from two well-characterized nuclear multiprotein complexes: the NuRD (nucleosome remodeling and deacetylase) complex and the SIN3A complex, also referred to as the Sin3A-HDAC complex (Figure 2B,C). The NuRD complex consists of HDAC1, HDAC2, CHD4, MTA1, MTA2, MBD2/3, RBBP4 (RbAp46), and RBBP7 (RbAp46), while the SIN3A complex comprises HDAC1, HDAC2, RBBP4 (RbAp46), RBBP7 (RbAp46), Sin3A, and SAP18. Notably, both complexes are well-documented in the literature as transcriptional co-repressor complexes that recruit HDAC1/2 [54]. The heatmap presented in Figure 2D illustrates the enrichment fold changes (log2) for the individual members of these two complexes, demonstrating consistent enrichment of these proteins across both IP conditions. Furthermore, Figure 2E provides a comprehensive overview of the label-free quantitation intensities (LFQ) and sequence coverage percentages (SC%) for each identified protein. Notably, RbAp48 (RBBP4) exhibited the highest SC%, while Sin3A demonstrated the lowest SC%. This discrepancy can be attributed to the relatively high molecular weight of Sin3A, which is 145 kDa, in contrast to RbAp48 (RBBP4), which has a molecular weight of only 48 kDa. This list does not contain HDAC1 and HDAC2, as we have previously reported their LFQ and SC% in our earlier report [43].

Together, the consistent co-purification of NuRD- and SIN3A-associated proteins across independent IP-MS strategies identifies IFI16 as a potential scaffold for these two HDAC1/2-recruiting chromatin-modifying complexes.

### 3.4. IFI16-NuRD and IFI16-Sin3A Complexes Localize with the KSHV Genome

**Figure 3:**
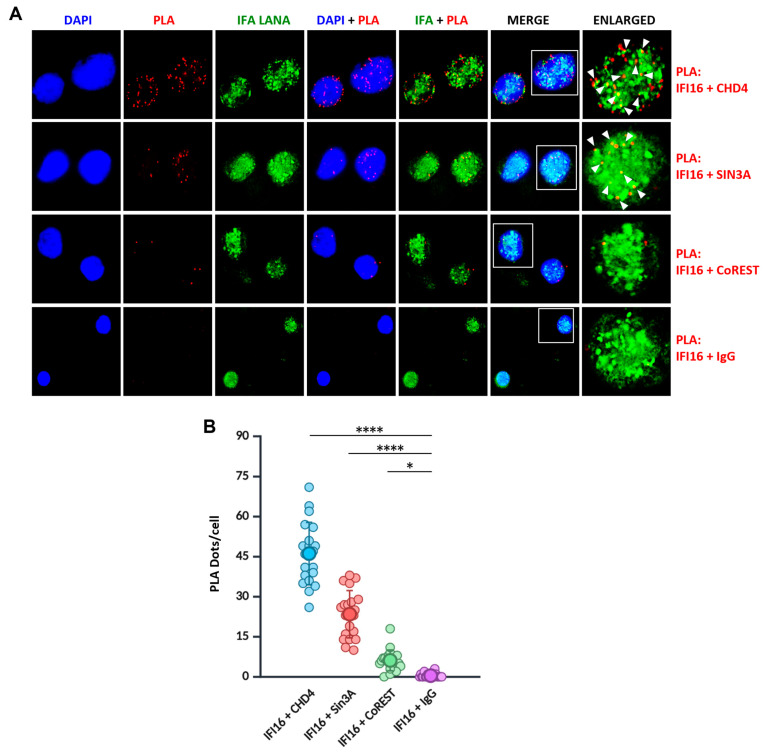
Interaction of IFI16 with CHD4 and Sin3A. (**A**) Proximity ligation assay (PLA, red) was performed for CHD4, Sin3A, CoREST, and IgG with IFI16 in BCBL-1 cells. The red channel shows the PLA puncta. Also, IFA (green) for KSHV LANA was conducted in the same experiment. White arrowheads in the enlarged image point to the colocalization (yellow) between the PLA dots and KSHV LANA. (**B**) The quantification of the PLA dots is represented as PLA dots/cell in a swarm plot. Kruskal–Wallis test with Dunn’s multiple comparisons test was performed (BioRender.com). *p* < 0.05 (*), *p* < 0.0001 (****).

Next, to confirm the interaction between IFI16 and the NuRD and Sin3A complexes, we employed proximity ligation assays (PLA), a technique that generates a fluorescent signal when two interacting proteins are in close proximity (approximately 40 nm). Our analysis specifically targeted IFI16’s interaction with key representatives from both the complexes: CHD4 from the NuRD complex and Sin3A from the Sin3A complex. The results demonstrated a robust interaction between IFI16 and both CHD4 and Sin3A, as evidenced by strong PLA signals detected between these proteins (Figure 3A,B). To ensure the specificity of the observed interactions, we performed a control PLA using IgG under similar conditions. Furthermore, we performed an additional control by conducting PLA between IFI16 and CoREST, another well-characterized HDAC1/2-recruiting transcriptional co-repressor complex found in mammalian cells. Consistent with our IP-LFQ-MS data, which did not identify CoREST as a binding partner of IFI16, we observed no significant PLA signal between IFI16 and CoREST, thereby corroborating our MS findings.

In conjunction with the PLA experiment, we also conducted immunofluorescence assays (IFA) targeting the KSHV latency-associated nuclear antigen (LANA) in the same experiment. LANA is considered a representative marker of the KSHV episomal genome due to its persistent association with the KSHV episome during latency [21]. This approach allowed us to investigate the potential colocalization of the IFI16-NuRD and IFI16-Sin3A complexes with the KSHV genome. Interestingly, our findings demonstrated that both PLA signals corresponding to IFI16-CHD4 and IFI16-Sin3A robustly colocalized with LANA puncta (Figure 3A, white arrowheads), strongly supporting the potential role of IFI16 in the recruitment of these co-repressor complexes to the KSHV chromatin.

### 3.5. Recruitment of HDAC1 to the NuRD and the SIN3A Complexes Is Dependent on the Presence of IFI16

**Figure 4:**
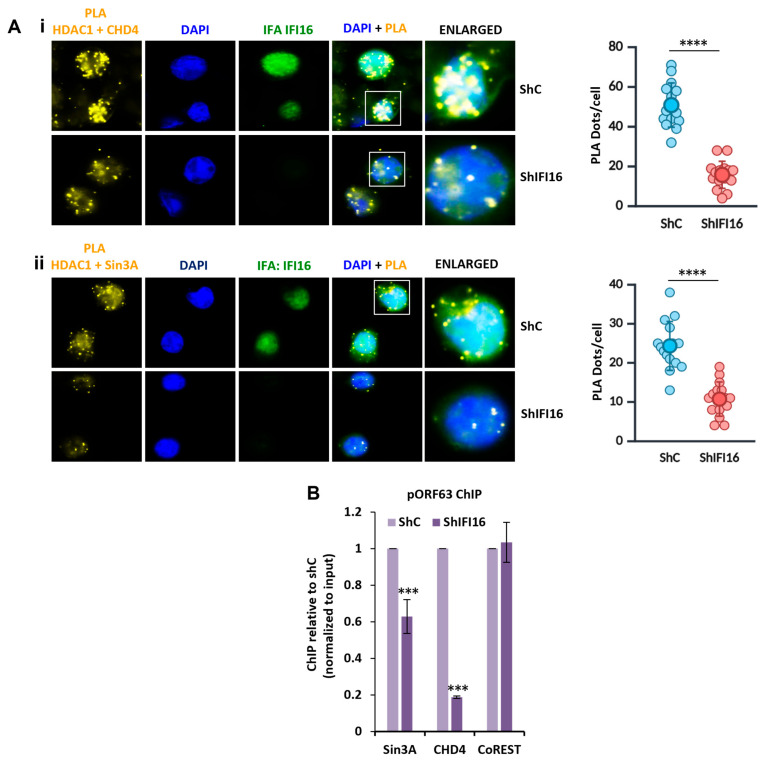
IFI16’s role in the interaction between HDAC1 and the NuRD and Sin3A complexes. (**A**) IFI16 was knocked down in BCBL-1 using shIFI16 lentiviral transduction. Control (shC) and KD (shIFI16) cells were subjected to PLA (yellow) for CHD4 (**i**) and Sin3A (**ii**) with HDAC1. IFA (green) for IFI16 shows IFI16 KD efficiency. A swarm plot for the corresponding PLA panels represents the number of PLA dots/cell. (**B**) The recruitment of chromatin remodeling complexes, NuRD, Sin3A, and CoREST, to the ORF63 promoter was studied by ChIP using anti-Sin3A, anti-CHD4, and anti-CoREST antibodies, respectively. Error bars represent mean ± standard deviation (SDEV) from three independent biological replicates. Statistical significance was determined by unpaired two-tailed Student’s *t*-test; *p* < 0.001 (***), *p* < 0.0001 (****).

Both the NuRD and Sin3A transcriptional co-repressor complexes depend on the deacetylase function of HDAC1 to compact chromatin and suppress transcription. Consequently, we next investigated the interaction between HDAC1 and the NuRD and Sin3A complexes following the KD of IFI16. Using a similar experimental setup to that outlined in Figure 1A, we performed PLA between HDAC1 and CHD4 and Sin3A in BCBL-1 cells. Our data indicated that after IFI16 KD depletion, the number of PLA dots for HDAC1-CHD4 and HDAC1-Sin3A significantly decreased compared to shC (Figure 4A). This suggests that IFI16 may act as a stabilizer for HDAC1 within these two complexes.

### 3.6. IFI16 Plays a Role in Recruiting the NuRD and the Sin3A Complexes to KSHV Promoters

Subsequently, we also investigated the role of IFI16 in recruiting the NuRD and Sin3A complexes to the KSHV promoters. To address this, we conducted ChIP analyses for CHD4, Sin3A, and CoREST following the KD of IFI16 in BCBL-1 cells. The results demonstrated that the depletion of IFI16 led to a significant reduction in the recruitment of both Sin3A and CHD4 to the ORF63 promoter (Figure 4B). However, consistent with our observations in Figure 3, IFI16 KD did not affect the recruitment of CoREST under similar conditions.

## 4. Discussion

Histone deacetylases (HDACs) play a pivotal role in the epigenetic regulation of herpesviruses, including KSHV, influencing both the establishment and maintenance of latency [59,60]. During latency, KSHV episomes are tightly associated with host nucleosomes, which are marked by various repressive histone modifications, including hypoacetylation. Previous reports indicate that HDACs, particularly HDAC1 and HDAC2, are recruited to KSHV chromatin, which contributes to the maintenance of a condensed chromatin state that silences lytic gene expression and enforces latency [24,33,61]. HDAC inhibitors (HDACi), such as Valproic acid (VPA), trichostatin A (TSA), nicotinamide, sirtinol, tubacin, sodium butyrate (NaB), and suberoylanilide hydroxamic acid (SAHA), have been shown to disrupt this silencing, leading to the activation of the viral lytic switch gene ORF50/RTA and thereby inducing reactivation of the virus [28,59,62]. Thus, the dynamic regulation of histone acetylation by HDACs constitutes a critical epigenetic switch that governs the KSHV life cycle, with significant implications for viral pathogenesis and therapeutic targeting.

The recruitment of HDAC1 and HDAC2 to chromatin is a highly regulated process predominantly achieved through their incorporation into multi-protein repressive complexes, rather than through direct binding to DNA. These HDACs function as essential components of various chromatin-modifying complexes, such as the NuRD, Sin3A, and CoREST, which direct HDAC1 and HDAC2 to specific genomic loci [63]. Recently, in a siRNA screen of host epigenetic factors essential for regulating KSHV latency, Naik et al. identified the NuRD complex as a repressor of KSHV lytic genes both during de novo infection and the maintenance of viral latency [64]. Similarly, multiple studies have also reported the binding of Sin3A to the latent KSHV chromatin [65,66], suggesting a broader involvement of these complexes in regulating KSHV gene expression. While the roles of HDAC1, HDAC2, and the NuRD and Sin3A complexes in KSHV latency are relatively well characterized, the mechanisms by which these repressive complexes are directed to the KSHV genome remain poorly understood.

Interestingly, despite the presence of RbAp46 and RbAp48, two proteins that bind to histones, numerous studies have demonstrated that the NuRD and the Sin3A complex are unable to deacetylate chromatin-associated nucleosomal histones in vitro. In contrast, free core histones have been identified as effective substrates for this deacetylation process [67]. This indicates that additional DNA-binding factors are required to recruit the NuRD and Sin3A complexes to the target chromatin to deacetylate nucleosomal core histones [63]. One such Sin3 recruiting complex is the silencing mediator for retinoid and thyroid receptors/nuclear co-repressor (SMRT/NCoR) [68]. Likewise, evidence indicates that the recruitment of the NuRD complex also relies on external DNA-binding proteins rather than on the inherent DNA-binding subunits [63]. In this context, the zinc finger DNA-binding proteins IKZF1 and IKZF3 have been shown to associate with the NuRD complex in T cells and recruit it to transcriptionally silenced regions of the genome during lymphoid lineage determination [69]. Our findings here suggest that IFI16 is another such factor that plays a significant role in recruiting the NuRD and Sin3A complexes to the KSHV chromatin. Notably, KSHV LANA has been shown to interact with Sin3A, which is suggested to facilitate the latter’s recruitment to the KSHV genome [66,70,71]. Furthermore, we have previously demonstrated that IFI16 interacts with LANA in latently infected cells [43], which raises the intriguing possibility that these proteins, along with their associated complexes, engage in cooperative interactions to form a larger multiprotein complex that regulates the epigenetic silencing of KSHV during latency.

IFI16 has garnered increasing attention not only as a DNA sensor but also as a chromatin-associated regulatory scaffold capable of recruiting various epigenetic repressors to chromatin [23,31,34]. Interestingly, in addition to its discussed role in viral epigenetics, recent studies not specifically focused on herpesviruses have elucidated that IFI16 also engages in physical interactions with components of the cellular epigenetic machinery, suggesting its broader role in cellular epigenetic pathways. Specifically, Kang et al. have reported the pull-down of IFI16 during IP of MTA-1, a critical scaffolding component of the NuRD complex, proposing that the MTA1-IFI16 complex may play a significant role in the epigenetic regulation of estrogen receptor alpha (ERα) expression in breast cancer [72]. Similarly, another study identified the IFI16-MTA-1 complex within the context of aggressive ERα-negative breast cancer [73]. Collectively, these findings position IFI16 as a molecular bridge linking innate immune sensing with chromatin remodeling, thereby facilitating the epigenetic silencing of viral genomes, such as KSHV, during latency.

Our data indicate that among the four core histone subunits, IFI16 most significantly mediates the deacetylation of H2B. While the acetylation of histones H3 and H4 is more widely established as regulators of cellular transcription, H2B acetylation is increasingly being recognized for its critical role in transcription initiation and elongation, particularly within the promoter and enhancer regions of actively transcribed genes [74]. Moreover, H2B acetylation serves as a recruitment signal for other bromodomain-containing proteins and chromatin remodelers, which are essential for facilitating RNA polymerase II progression [75,76]. This presents a compelling hypothesis suggesting that IFI16-mediated acetylation of histone H2B on KSHV chromatin serves as a dynamic regulatory mechanism that intricately modulates the expression of KSHV lytic genes. Importantly, our prior studies have demonstrated that the IFI16 protein undergoes selective degradation during the lytic reactivation of cells harboring latent KSHV [35]. Therefore, during the latency phase, when IFI16 levels remain intact, the protein facilitates the recruitment of the NuRD and Sin3A complexes, resulting in the deacetylation of KSHV lytic promoters and effectively silencing their expression. However, upon the induction of lytic reactivation, the targeted degradation of IFI16 occurs, thereby alleviating the hypoacetylation pressure on the lytic promoters and allowing for their derepression.

This study raises several important questions that merit further investigation. First, it remains to be determined whether IFI16 functions as a stable, resident component of the NuRD and Sin3A co-repressor complexes in broader cellular contexts or whether its incorporation into these complexes is conditional, specifically mobilized during antiviral responses such as herpesviral gene silencing. Second, considering IFI16’s established role as a nuclear DNA sensor, it will be intriguing to explore the crosstalk between the innate immune pathways and the epigenetic restriction factor (eRF) roles of IFI16 [23]. Third, it will be worthwhile to explore whether KSHV exploits this IFI16-NuRD/Sin3A axis to establish and maintain its latency by promoting the epigenetic silencing of lytic promoters. Fourth, it is unclear whether this mechanism is unique to KSHV or represents a broader strategy employed across the herpesvirus family; determining whether IFI16 mediates NuRD/Sin3A recruitment to chromatin in the context of other herpesviruses could offer valuable insight into conserved host–virus epigenetic interactions. Finally, future studies can investigate whether post-translational modifications of IFI16, such as acetylation or phosphorylation, modulate its ability to recruit or stabilize these co-repressor complexes at viral chromatin. Collectively, future studies to address these questions will enhance our understanding of how host nuclear surveillance pathways intersect with chromatin remodeling machinery to control viral latency and reactivation.

## Data Availability

All data needed to evaluate the conclusions in the paper are present in the manuscript.
